# Metal-encapsulated organolead halide perovskite photocathode for solar-driven hydrogen evolution in water

**DOI:** 10.1038/ncomms12555

**Published:** 2016-09-06

**Authors:** Micaela Crespo-Quesada, Luis M. Pazos-Outón, Julien Warnan, Moritz F. Kuehnel, Richard H. Friend, Erwin Reisner

**Affiliations:** 1 Christian Doppler Laboratory for Sustainable SynGas Chemistry, Department of Chemistry, University of Cambridge, Cambridge CB2 1EW, UK; 2 Department of Physics, University of Cambridge, Cambridge CB3 OHE, UK

## Abstract

Lead-halide perovskites have triggered the latest breakthrough in photovoltaic technology. Despite the great promise shown by these materials, their instability towards water even in the presence of low amounts of moisture makes them, *a priori*, unsuitable for their direct use as light harvesters in aqueous solution for the production of hydrogen through water splitting. Here, we present a simple method that enables their use in photoelectrocatalytic hydrogen evolution while immersed in an aqueous solution. Field's metal, a fusible InBiSn alloy, is used to efficiently protect the perovskite from water while simultaneously allowing the photogenerated electrons to reach a Pt hydrogen evolution catalyst. A record photocurrent density of −9.8 mA cm^−2^ at 0 V versus RHE with an onset potential as positive as 0.95±0.03 V versus RHE is obtained. The photoelectrodes show remarkable stability retaining more than 80% of their initial photocurrent for ∼1 h under continuous illumination.

## Abstract (web summary)

Lead-halide perovskites are sensitive to humidity, which limits their use in water splitting applications. Here, the authors protect the perovskite layer with Field's metal, driving photoelectrocatalytic hydrogen evolution in an aqueous solution for approximately one hour under constant illumination.

## Graphical abstract

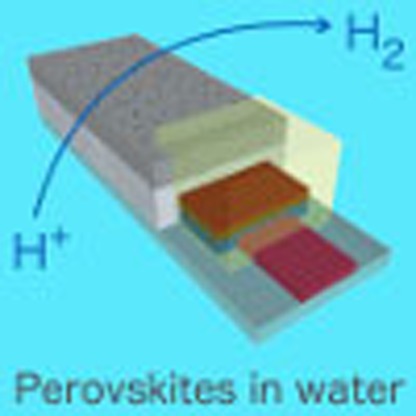

Society is facing a number of pressing issues including the depletion of the Earth's finite pool of fossil fuel resources, the steady increase of energy demand, and the predicted detrimental changes to the Earth's climate due to CO_2_ emission from the combustion of carbon-based fuels^1^. Photovoltaic (PV) technology is expected to play a major role covering future electricity needs, which accounts for ∼18% of the world's energy demand^2^. On the other hand, artificial photosynthesis, which utilizes sunlight to create solar fuels such as H_2_, is considered as a promising method for tackling the fuel demand (67% of world energy consumption relies on non-renewable fuels) in a post-fossil era^2^,^3^.

Hybrid organic–inorganic perovskites are the latest breakthrough as light harvesters in solar cells^4^. Intensive research has led to a rapid rise in power conversion efficiency (PCE) since their original publication in 2009 (ref. 5) to values reaching 20% in 2015 (refs 6, 7), making it the fastest-developing technology in the history of PV. The use of perovskites affords several advantages: a direct bandgap that is tuneable by changing the chemical composition of the material^8^,^9^, ambipolar charge transport^10^, long carrier lifetimes^11^ and long charge diffusion lengths in the micrometre range^12^,^13^,^14^. Perovskites, such as the most common lead-iodide based CH_3_NH_3_PbI_3_, constitute excellent candidates for photoelectrochemical H_2_ evolution since they also have a favourable band alignment for reducing protons, where, depending on the pH, the position of the conduction band could provide 100–500 mV of driving force.

Despite the great promise of perovskites, they still show one major drawback; they are inherently unstable in water. Indeed, it was found that the lattice structure of CH_3_NH_3_PbI_3_ can be easily broken in the presence of moisture, which is followed by the decomposition of the material into PbI_2_ (ref. 15). State-of-the-art protection techniques in PV cells include moisture-blocking hole transporters^16^,^17^, atomic layer deposition (ALD)^18^ or water-free ALD^19^ and hydrophobic carbon electrodes^20^,^21^.

However, these techniques are either unsuitable for preparing photocathodes since they were developed for standard n-i-p systems, or costly and difficult to scale-up. As a result, currently published work regarding perovskites for solar fuel production has involved one^22^,^23^ or two^24^ external standard perovskite solar cells wired to suitable catalysts inside the aqueous solution for water electrolysis. Systems with a perovskite immersed in water to directly photogenerate H_2_ are unknown.

In this work, we report the successful protection and integration of a hydrogen evolution catalyst (HEC) with a perovskite-based photocathode in a single device capable of stable photoelectrochemical H_2_ generation in water. We prepare photocathodes using CH_3_NH_3_PbI_3_ as light harvester following the standard inverse (or p-i-n) planar solar cell configuration. In order to protect the perovskite, we develop a novel and simple metal encapsulation technique using a fusible InBiSn alloy (Field's metal, FM) that protects the light absorber from water while simultaneously allowing the efficient transfer of photogenerated electrons to a Pt HEC deposited on its surface.

## Results

### Perovskite solar cell

**Figure 1:**
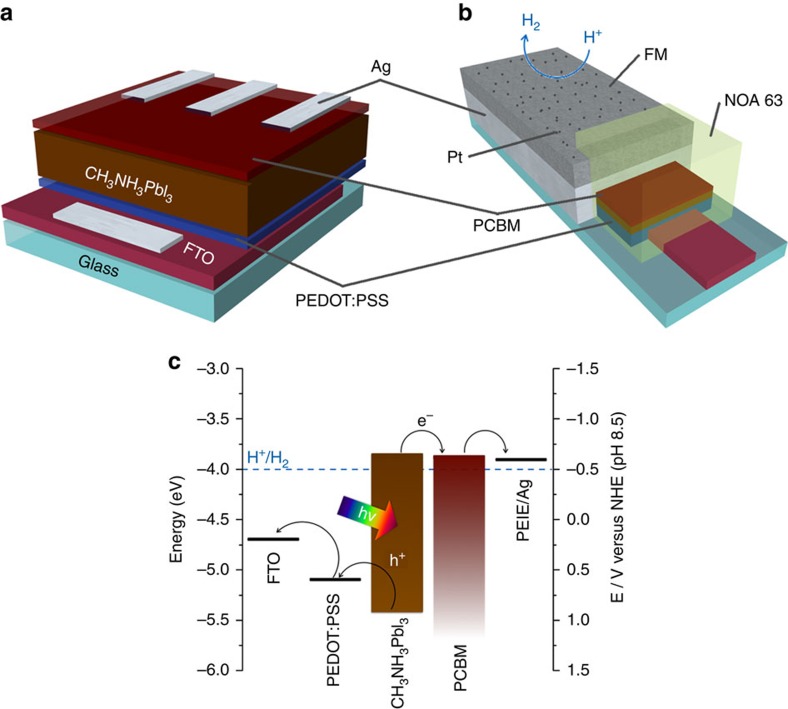
Material and electronic configuration of the perovskite-based solar cells and photocathodes. (**a**) Schematic representation of the structure of the perovskite solar cell. An inverted p-i-n configuration was used, with the general FTO/PEDOT:PSS/perovskite/PCBM/PEIE:Ag structure. (**b**) Scheme of the solar cell adapted as a photocathode for solar H_2_ production. The structure remains the same, but an extra metal-encapsulating layer of FM and Pt as a HEC are added on top of the Ag layer. (**c**) Energy diagram of both devices; for conversion between NHE and RHE reference electrodes, use *E*_RHE_=*E*_NHE_+0.059 × pH.

With the ultimate goal of protecting a perovskite-based photocathode and integrating a HEC, we firstly prepared perovskite solar cells (see Fig. 1a for a schematic representation and Fig. 1c for its energy diagram). We followed a typical inverted or p-i-n configuration, where the p-type hole collection layer is processed first on the transparent conducting glass^25^. We spin-coated a 40 nm thick poly(3,4-ethylenedioxythiophene) polystyrene sulfonate (PEDOT:PSS) layer as hole transporting material on fluorine-doped tin oxide (FTO)-coated glass, followed by a 300 nm thick CH_3_NH_3_PbI_3_ layer as light harvester and a 40 nm thick [6,6]-phenyl-C_61_-butyric acid methyl ester (PCBM) layer as electron transporting material (ETM), as confirmed by atomic force microscopy (Supplementary Fig. 1). A 100 nm thick film of Ag was evaporated to form the top contacts of the perovskite solar cell component. Ethoxylated polyethylenimine (PEIE) was spin coated on top of the ETM and under the Ag to adjust its work function, improving electron transfer from PCBM to Ag^26^.

In order to obtain a homogeneous perovskite layer we prepared the material using lead acetate as precursor^27^, which leads to ultra-smooth and pin-hole-free perovskite films^28^. The films were subsequently solvent-annealed at 100 °C in the presence of *N-N-*dimethylformamide (DMF) vapour to induce the growth of larger crystal domains in the material^29^.

Supplementary Fig. 2 shows the scanning electron microscopy images of the perovskite films under various magnifications. The films are homogeneous with total coverage and grain sizes of a few hundred nanometres. The ultraviolet–visible spectrum of the perovskite film in Supplementary Fig. 3a shows a broad absorption throughout the visible region. This translates into a large fraction of solar photons being absorbed^30^, which makes it a particularly efficient candidate for solar fuel production. The bandgap of the prepared films was estimated with a Tauc plot (Supplementary Fig. 3b) to be approximately 1.56 eV, in accordance with literature values^31^,^32^. The crystal structure of the perovskite film was analysed by powder X-ray diffraction (XRD; see Supplementary Fig. 4). The most prominent diffraction peaks at Bragg angles of 13.98° and 28.32° correspond to those from the (110) and (220) crystal planes of the tetragonal perovskite structure, respectively^33^. We also characterized the perovskite films alone and in conjunction with the transport layers through steady-state and time-resolved photoluminescence (PL) experiments as shown in Supplementary Fig. 5a. A PL emission maximum of the perovskite at 775 nm is in line with literature^34^. The PL decay of the perovskite is faster in the presence of either PEDOT:PSS or PCBM, and the fastest for the complete device (PEDOT:PSS/perovskite/PCBM) as seen in Supplementary Fig. 5b, indicating an efficient charge transfer from the perovskite to the electron and hole transporting materials^35^.

**Figure 2:**
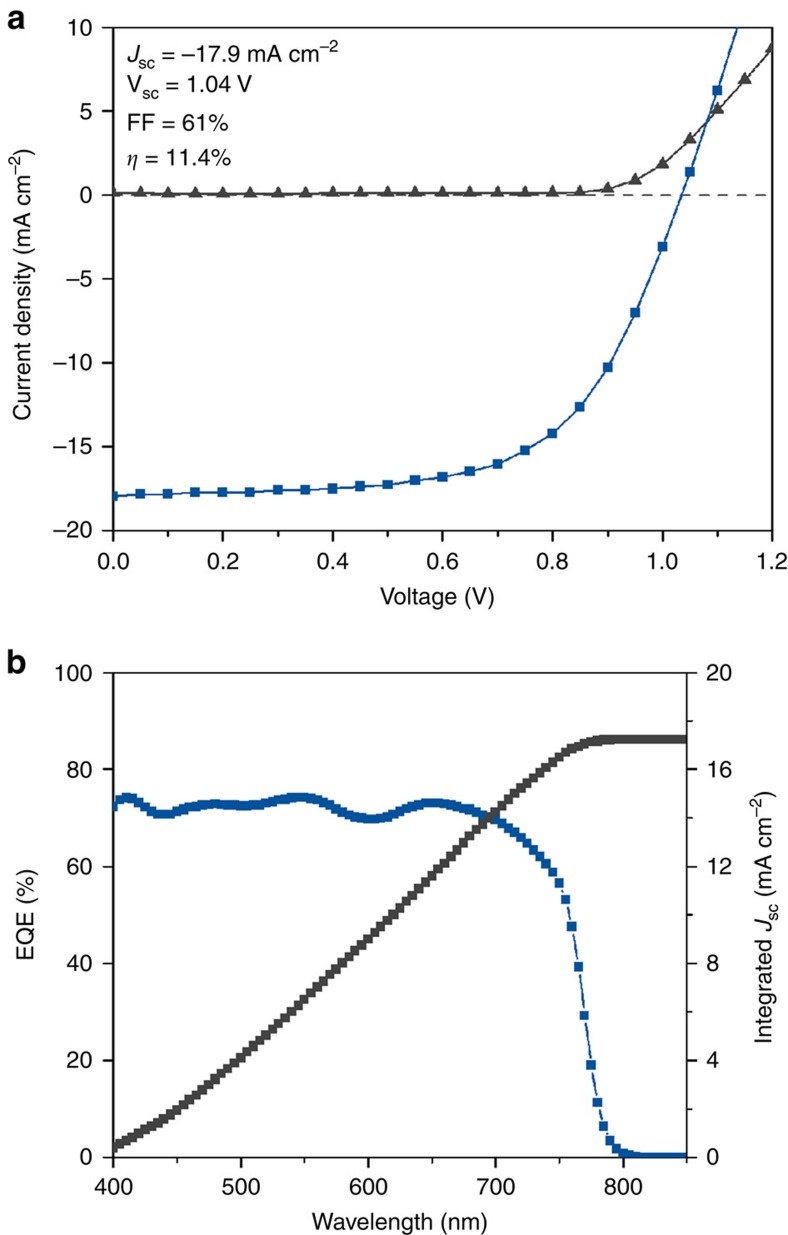
Perovskite solar cell performance. (**a**) Light (blue line and symbols) and dark (grey line and symbols) *IV* curves of our champion device under irradiation from a solar simulator and adjusted to AM 1.5 G illumination (100 mW cm^−2^, active area: 12 mm^2^, 0.05 V step and 50 ms dwell time, measured from reverse to forward bias). (**b**) EQE (blue line and symbols) and integrated *J*_sc_ (grey line and symbols) of the same device.

The perovskite solar cells were measured under simulated AM1.5G solar light (100 mW cm^−2^) and showed an average short circuit current (*J*_sc_), open circuit voltage (*V*_oc_), fill factor and PCE of: 15.0±1.4 mA cm^−2^, 1.0±0.09 V, 54.0±6.0% and 7.7±1.5%, respectively. The current–voltage (*IV*) curve of our champion device, the external quantum efficiency (EQE) and integrated *J*_sc_ for the same cell are shown in Fig. 2. These results are comparable with literature devices with the same configuration and precursor^36^,^37^.

The statistical analysis performed on the full set of measurements (Supplementary Fig. 6) can be found in the Supplementary Discussion. We found only a marginal hysteresis effect on the cells (Supplementary Fig. 7). The *IV* characteristics of the cells did not vary with the scan step size (Supplementary Fig. 8), and the effect of the mask size on the cell performance was small (Supplementary Fig. 9). The mismatch factor of around 10% between the solar simulator and AM1.5G solar spectra can also be seen in Supplementary Fig. 10.

### Protecting perovskites in water

The main drawback of perovskite materials is their inherent instability in water. CH_3_NH_3_PbI_3_ readily decomposes into PbI_2_ with concomitant dissolution of CH_3_NH_3_I when submerged in water^15^. A solution to this problem is the use of an indirect and two-component system, where the perovskite PV device is kept outside the electrolyte solution and is wired to a separate water electrolyzer to split water into H_2_ and O_2_ (refs 22, 23, 24).

An alternative and more direct solution would be the use of the perovskite PV unit in a single component system, where light absorption is coupled to HEC without external wiring and intermittent generation of electricity. This challenging goal requires immersion of the perovskite in water and can only be accomplished with a suitable layer to protect the perovskite and conduce the photogenerated electrons to the HEC at the electrode–solution interface. Furthermore, the material must be processable at temperatures below 100 °C, which is the thermal stability limit for CH_3_NH_3_PbI_3_ (ref. 15). This eliminates the previously reported protection techniques except ALD, but this approach is complex, costly and has not been proven successful protecting perovskites. The only reports on submerged perovskites use a protective layer of evaporated Ni that oxidizes Na_2_S as a hole scavenger (this device loses 80% of its initial photocurrent after 15 min of operation^38^) or a pinhole-free mixture of PMMA/CNT/P3HT covered with Ni used for water oxidation^39^.

We therefore focused our attention on a novel protection methodology and designed a metal-based encapsulation technique to use perovskite solar cells as the basis for photocathodes. We identified FM as an encapsulating candidate as it is a non-toxic eutectic alloy of bismuth (32.5%), indium (51%) and tin (16.5%) that melts at 62 °C.

A layer of FM was deposited on top of the Ag-covered sections of the photocathode with the dual aim of protecting the perovskite from water while efficiently shuttling the photogenerated electrons to the surface, where the hydrogen production reaction takes place. Figure 1b shows a schematic representation of the photocathode, and a detailed description of the preparation procedure can be found in the ‘Methods' section. Briefly, a solid piece of FM was placed on the Ag-covered area of the photocathode, and placed on a hot plate. The device was subsequently heated to 70 °C until all FM had melted. In this case, the purpose of evaporating Ag was twofold. First, it provided a good interface with PCBM, thus improving electron injection into the overlying FM layer. Second, the high wettability of Ag by the FM under N_2_ (as compared with the low wettability of all the other layers) allowed the precise confinement of FM to the desired area, thus facilitating preparation and avoiding short-circuits in the system. The device was allowed to cool down to room temperature and the edges were sealed with ultraviolet-curing resin (NOA 63).

The water resistance of the metal-encapsulated photocathode was first confirmed by placing it in an aqueous buffer solution (0.1 M borate, pH 8.5) for several hours and an unprotected photocathode (that is, without the Ag and FM layers) was also studied for comparison. While the unprotected photocathode was completely degraded within 1 min, the protected photocathode only began to show faint signs of degradation after 6 h submerged in the absence of light and electrochemical bias (Supplementary Fig. 11).

### Electrocatalytic H_2_ production on FM

Supplementary Fig. 12 shows the cyclic voltammogram of pure FM in an aqueous solution (0.1 M borate, pH 8.5) in a two-compartment electrochemical cell with a Ag/AgCl reference and Pt mesh counter electrode. The redox waves present on the FM are probably due to redox processes assigned to the alloy matrix and no catalytic current was observed in the absence of a HEC. Therefore, Pt as a benchmark HEC was introduced by an electroless deposition by immersing the FM into an aqueous solution of K_2_PtCl_4_ (5.2 mM; *E*^0^=0.75 V versus SHE^40^) for 1 min. Metals that form FM can easily be oxidized by Pt^2+^, resulting in a homogeneous deposition of metallic Pt on the surface of the FM without the requirement of light, an applied potential, or a sacrificial reagent. XRD spectra recorded before and after the platinization step (Supplementary Fig. 13) show a broad peak at a Bragg angle of 39.7° consistent with the main diffraction peak of Pt (111) (JCPDS ICDD card 88–2343). A crystallite size of ∼4.5 nm was determined for the Pt nanoparticles from the full width at half maximum (FWHM) of this peak by applying the Scherrer equation. The ability of the platinized FM to reduce protons is demonstrated by the catalytic onset potential close to 0 V versus RHE in an aqueous borate solution (0.1 M, pH 8.5) in the cyclic voltammogram shown in Supplementary Fig. 12. The use of borate buffer close to pH neutral conditions enables the coupling of a photocathode with a state-of-the art photoanode material such as BiVO_4_ (ref. 41), which dissolves under extreme pH values^42^. Operation at low pH would not be feasible due to the oxidation of the constituent metals in the FM, whereas operation under basic conditions also displays good activity and expands the general applicability of the FM protection approach (Supplementary Fig. 14).

### Solar H_2_ production with protected perovskite photocathode

**Figure 3:**
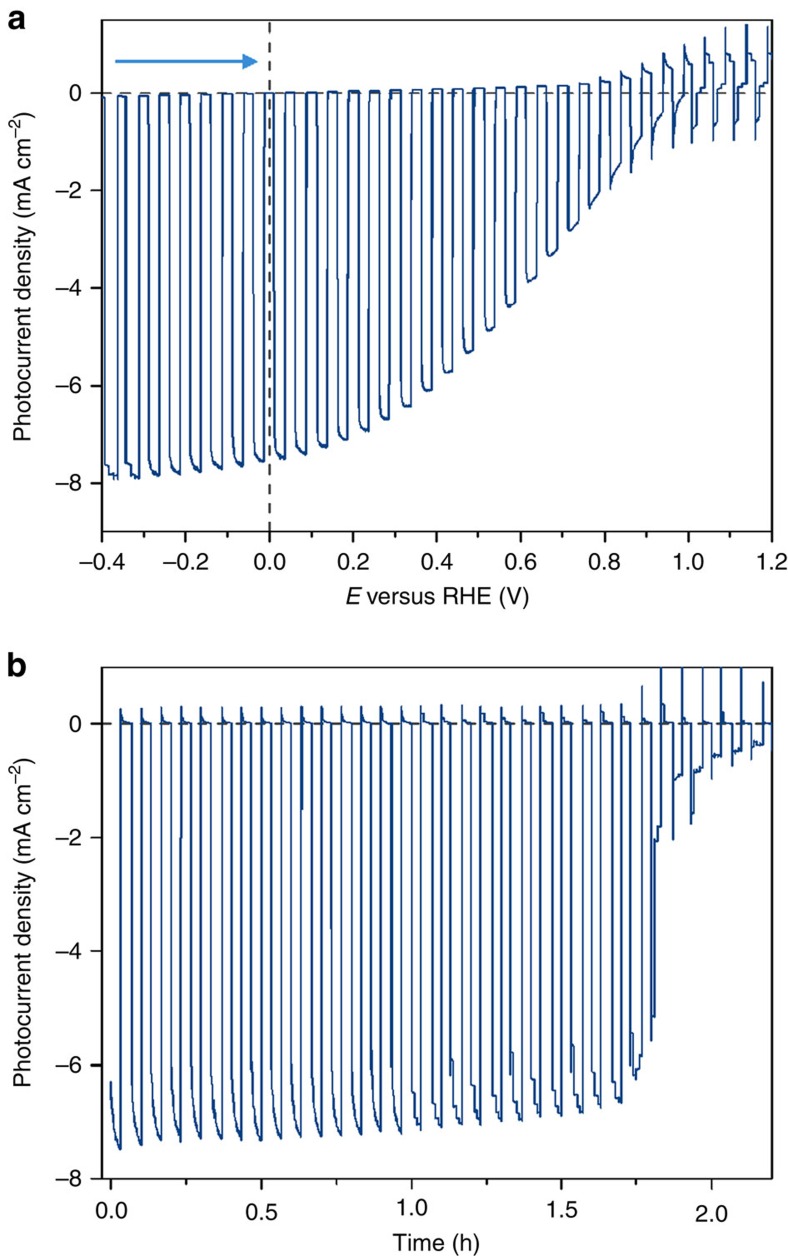
Perovskite photocathode performance during H_2_ photogeneration. (**a**) Typical linear sweep voltammetry of the perovskite-based photocathode at a scan rate of 5 mV s^−1^. The arrow denotes the scan direction. (**b**) Chronoamperometric trace recorded at an applied potential of 0 V versus RHE. An aqueous buffer solution (0.1 M borate, pH 8.5), chopped solar light irradiation (AM 1.5 G, 100 mW cm^−2^, *λ*>400 nm) and an inert (N_2_) atmosphere at room temperature were used in both experiments.

The photocathodes were studied in a three-electrode configuration with both the perovskite working and an Ag/AgCl reference electrode separated from the Pt mesh counter electrode by a Nafion membrane. Figure 3a shows a typical linear sweep voltammogram (LSV) in aqueous electrolyte solution (0.1 M borate, pH 8.5) during chopped simulated solar light irradiation (AM 1.5 G filter, 100 mW cm^−2^, *λ*>400 nm) recorded at 5 mV s^−1^. The average photocurrent density obtained at 0 V versus RHE was −6.9±1.8 mA cm^−2^, with a record device at −9.8 mA cm^−2^. A video of an actual device under operation at 0 V versus RHE can be seen in Supplementary Movie 1. The average onset potential obtained was 0.95±0.03 V versus RHE. Since no direct liquid–semiconductor junction is formed, the onset potential is related to the cell photovoltage, or *V*_oc_. Indeed, both values are strikingly similar, suggesting only small losses at the Ag/FM junction. The very positive onset potential makes this photocathode highly attractive for future tandem water splitting devices.

Linear sweep voltammograms (LSVs) performed without a *λ*<400 nm cut-off filter (Supplementary Fig. 15a) show that, as expected from the ultraviolet–visible spectrum, most of the photocatalytic response proceeds from the light absorbed by the perovskite in the visible region of the solar spectrum. The scan direction influences the photocurrent slightly (Supplementary Fig. 15b), which is consistent with the small hysteresis observed in the solar cells.

The photocathodes were stable under operation conditions for more than 1.5 h in all cases, retaining more than 80% of the initial photocurrent as studied by light-chopped chronoamperometry (Fig. 3b). The slow rise in photocurrent upon irradiation observed in the chronoamperometry traces is also known in perovskite solar cells. It was proposed that irradiation of lead-iodide perovskite triggers the migration of iodide, which in turn leads to a decrease in the density of trap states^43^, and a slow increase in PL over time. This phenomenon might also be responsible for the slow increase in photocurrent in our experiments. Gas samples were taken from the cathodic headspace at the end of each chronoamperometric experiment and analysed by gas chromatography. The Faradaic efficiency (FE) of the photocathodes was found to be 95.1±2.2 %, confirming that the photocurrent observed corresponded to H_2_ generation. These are striking results considering the well-known instability of lead-halide perovskites in water^25^. An additional advantage of our metal encapsulation technique is that the layers of FM could be easily detached from the degraded photocathodes, polished, and reused several times.

Our perovskite-based photocathodes are superior to other hydrogen evolution materials when considering the obtained current density and onset potential. While p-Si can deliver higher photocurrents, it must be noted that research on that material as photocathode has a much longer history^44^ than that of perovskites. The reported perovskite-based photocathode shows comparable photocurrent densities to benchmark p-GaInP_2_ photoelectrodes^45^, and Cu_2_O-based photocathodes^46^,^47^. In all those cases, the materials were protected with expensive and time-consuming ALD techniques, while the method developed in this work is much simpler and more scalable. The major advantage of our perovskite photocathode is the much more anodic onset potential. Indeed, the perovskite photocathode presented here showed onset potentials of around 1 V versus RHE, which is ∼500 mV more positive that of the aforementioned photocathodes^44^. This makes our device very attractive for application in a tandem photoelectrochemical cell for overall solar water splitting. The non-transparency of the FM layer would not impede this application, since the photocathode would be, in such a case, the back electrode of the tandem system and the metallic coating could even be beneficial to reflect the light back to the perovskite.

In the present study, the exact time to full degradation varied depending on the photocurrent density given by the photocathode (Supplementary Fig. 16). In all cases, a sudden deactivation occurred when the degradation front (which was visible from the back of the electrode) reached the area of the perovskite over the FTO. The degradation was faster than in the dark and without applied bias (Supplementary Fig. 11). Furthermore, operation under continuous illumination (Supplementary Fig. 17) shows that indeed the total charge passed through the device seems to be a key element of the degradation. Thus, two hypotheses seem likely to explain the degradation. First, silver may be corroding when in contact with the halide ions forming silver halides in humid environments^48^,^49^. Second, the application of even a weak electrical field has recently been found to result in a rapid (hours) degradation of the material to PbI_2_ in the presence of moisture^50^. In both cases, the presence of water has been described as necessary in the literature, and is indeed confirmed in our case, since degradation always starts from the edges of the perovskite, but never in the centre of the film. An extended chronoamperometry experiment with platinized FM is shown in Supplementary Fig. 18. The FM/Pt electrode can perform electrocatalytic proton reduction at −0.1 V versus RHE (same buffer as the photoelectrochemical experiments) without catastrophic degradation, ruling out the stability of the FM/Pt being the limiting factor.

Nevertheless, we are confident that using more efficient perovskites and optimizing the top charge collection layer could further improve both the activity and stability of the photocathodes presented. Furthermore, our approach is very simple to apply, making it versatile enough for its application on a variety of unstable or photocorrodible materials.

**Figure 4:**
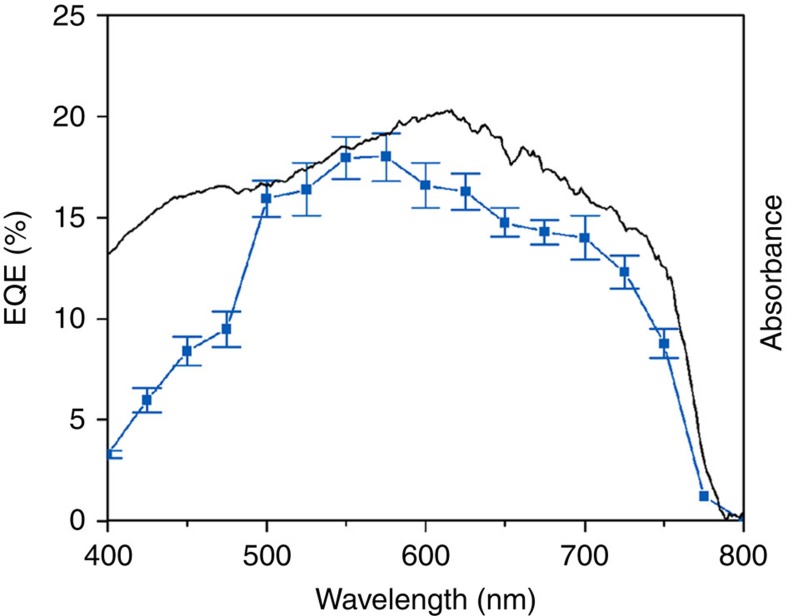
Effect of light wavelength on H_2_ generation with perovskite photocathodes. Overlay of the absorption (ultraviolet–visible) spectrum (grey line) of the perovskite film and the EQE (blue line and symbols) obtained with the perovskite photocathode in an aqueous buffer solution (0.1 M borate, pH 8.5) with monochromatic light (15 nm FWHM band-pass filters) and inert (N_2_) atmosphere at room temperature and 0 V versus RHE. The error bars denote the standard deviation of three different repetitive measurements in two different samples.

Finally, we used monochromatic light to measure the photocurrent produced at different wavelengths (FWHM of 15 nm) as shown in Fig. 4. The EQE of the system at 0 V versus RHE matches the ultraviolet–visible spectrum of the perovskite closely (which is overlaid in the same graphic), suggesting that the perovskite can efficiently generate hydrogen when coupled with a suitable HEC throughout the entire visible region of the solar spectrum.

In conclusion, a simple and scalable technique to simultaneously protect a perovskite PV component and couple it directly with a HEC in a single device to stably generate hydrogen in aqueous media has been presented. We employed a 7.7±1.5% PCE p-i-n configuration solar cell and developed a simple method to protect the perovskite from water in order to employ them as photocathodes for the hydrogen evolution reaction. A layer of FM was used as a protecting and conducting layer, capable of shielding the perovskite from water and allowing the transport of the photogenerated electrons to the top of the device, where they could reach the HEC and produce hydrogen. The average photocurrent density obtained at 0 V versus RHE was −6.9±1.8 mA cm^−2^, with a record device at −9.8 mA cm^−2^ and onset potentials as positive as 0.95±0.03 V versus RHE. This performance is superior to current benchmark systems, making these devices extremely interesting for application in overall tandem water splitting devices. The photocathodes retained 80% of their initial photocurrent for more than 1.5 h in aqueous solution under chopped light, and approximately 1 h under continuous illumination. This metal-encapsulation technique is simple and potentially also applicable to other types of unstable or photocorrodible materials.

Our work demonstrates that the high potential that perovskites have shown in the solar cell field can indeed be translated into artificial photosynthesis research. These findings will consequently also spur further attempts to bridge the optoelectronics and solar fuels communities, to find joint applications towards the ultimate goal of harnessing the power of the sun.

## Methods

### Methylammonium iodide synthesis

Hydroiodic acid (24.6 ml, 57% in H_2_O, Sigma) was added dropwise to 27.86 ml of methylamine (33% in absolute ethanol, Sigma) under N_2_ atmosphere with stirring at 0 °C for 2 h. The solvent was evaporated with a rotary evaporator at 50 °C and the obtained powder was dissolved in ethanol and precipitated by the addition of diethyl ether. The powder was washed twice with diethyl ether and dried in a vacuum oven at 60 °C overnight.

### Solar cell fabrication

FTO 14 × 14 mm glass (TEC 7, ∼7 Ω sq^−1^, Sigma-Aldrich) was used as a conducting substrate for the solar cells. Overall, 4 mm of FTO were etched of the glass substrate using Zn and 2 M HCl. Subsequently, the slides were washed with water, sonicated in isopropanol for 20 min, followed by sonication in acetone for 20 min, treated for 20 min with Ultraviolet/Ozone cleaning (Bioforce Nanosciences) and dried in air. Subsequently, PEDOT:PSS (AI 4083, Clevios) filtered through a 0.2 μm PES filter was spin-coated at 4,000 r.p.m. for 60 s and then heated at 120 °C for 20 min in air. The FTO-glass slides coated with PEDOT:PSS were then transferred into an anhydrous N_2_-filled glovebox. The perovskite active layer was prepared following a modification of the protocols reported elsewhere^29^,^51^. The perovskite film was prepared from a DMF (99.9%, Sigma) solution containing methylammonium iodide (3.3 M) and Pb(CH_3_CO_2_)_2_ (1.1 M, 99.999%, Sigma). The resulting solution was coated onto the FTO/PEDOT:PSS at 5,000 r.p.m. for 30 s. The substrates were kept at 30 °C for about 10 min, after which they were heated to 100 °C (6 °C min^−1^) in the glovebox, and a petri dish was placed upside down over them (10 μl of DMF were placed under the edge of the petri dish). The substrates were subsequently annealed for 45 min under the thus created DMF atmosphere and then left to cool to room temperature. The ETM consisted of PC_61_BM (99.5%, Solenne) layer spin coated from a 35 mg ml^−1^ in chlorobenzene solution at 3,000 r.p.m. for 45 s. A 0.2 wt% of PEIE (80% in H_2_O, Sigma) in isopropanol was spin coated at 3,000 r.p.m. for 30 s. Finally, a 100 nm thick layer of Ag was deposited on top of the ETM by thermal evaporation.

### Photocathode fabrication

The photocathodes were fabricated following the same procedure as described for the solar cells, only that the FTO, CH_3_NH_3_PbI_3_ and Ag patterns were designed to maximize the protection of the perovskite as shown on Fig. 1. Briefly, the FTO was etched from the surface of the glass slides to leave only a centred rectangular feature. PEDOT:PSS, CH_3_NH_3_PbI_3_, PCBM and PEIE were deposited on the slides following the same procedure described for the solar cells, but a 1 cm area of the glass was left uncoated (where FTO will subsequently be contacted). A 100 nm Ag layer was evaporated on top of the deposited materials leaving a few mm of them uncovered to avoid a direct contact between the FTO and Ag. A piece of FM corresponding to 1.5 g_FM_ cm^−2^_Ag_ was placed on the Ag-covered part of the photocathodes and heated to 70 °C inside of the glovebox. Once the FM melted completely, wetting only the parts of the device covered with Ag, the device was left to cool on the hot plate. No correlation was found between FM thickness and performance, but a minimum amount of 1.5 g_FM_ cm^−2^_Ag_ was used in order for the former to properly wet the entire Ag surface and avoid strains during preparation that could lead to device shorting. The edges of the photocathodes were protected with ultraviolet-curing resin (NOA 63, Norland) and the active area was ∼0.4 cm^2^. The surface of the FM was polished, cleaned with acetone and isopropanol before submerging the device in a 5.2 mM aqueous solution of K_2_PtCl_4_ (98%, Sigma) for 1 min. The photocathode was rinsed with water and dried in air. After using the photocathode, the resin was removed and the FM layer was detached using a scalpel. The top and bottom of the FM layer were polished to remove Pt and Ag residues, and reused on another fresh photocathode.

### Optical experiments

*IV* characteristics were measured in the dark and simulated solar irradiation (Oriel 92250A) using a Keithley 2636A source-measure unit. The current from the solar cell was compared with the current of a NIST-traceable calibrated photodiode (Thorlabs SM05-CAL). Both the device and the calibration cell were measured against a reference diode (SM05) to account for changes in light intensity between the measurements.

### Photoelectrochemical H_2_ evolution

Photoelectrochemical measurements were carried out on an electrochemical workstation (IVIUMSTAT) under inert atmosphere and at room temperature. A conventional three-electrode configuration was used with the perovskite, Pt mesh and Ag/AgCl/KCl_(sat)_ as working, counter and reference electrode, respectively, in an aqueous electrolyte solution (0.1 M borate, pH 8.5). In order to study the electrochemistry of FM, we contacted a flat piece of FM to a stainless steel rod with the use of Cu tape. After insulating the Cu/FM contact area, the exposed FM was polished, cleaned and plated with Pt through electroless deposition. In photoelectrochemical H_2_ evolution experiments, the working electrode was illuminated from the back with a 100 mW cm^−2^ solar light simulator (Newport Oriel, 150 W) equipped with an AM 1.5 G filter and an IR water filter. All electrochemical potentials are reported against the reversible hydrogen electrode (RHE) by using the equation *E* (V versus RHE)=*E* (V versus Ag/AgCl)+0.059 pH+0.197 (ref. 52). Hydrogen detection was carried out by gas chromatography by taking a 50 μl sample from the headspace of the photoelectrochemical cell at the end of chronoamperometry. Gas chromatography was carried out on an Agilent 7890A gas chromatograph using a HP-5 column (0.32 mm diameter) at 45 °C and N_2_ carrier gas with a flow rate of ∼3 ml min^−1^ and a thermal conductivity detector. Methane (2% CH_4_ in N_2_) was used as internal standard after calibration with different mixtures of known CH_4_/H_2_ compositions. The FE was determined by comparing the amount of hydrogen detected with the charge passed through the working electrode according to the equation:


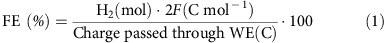


### Wavelength-dependent photon to current conversion

The photocathodes were irradiated by a solar light simulator (LOT LSN 254) equipped with a monochromator (LOT MSH 300) that was used to focus a single wavelength (accurate to a FWHM of 15 nm). Chronoamperometry was performed at 0 V versus RHE (PalmSens Emstat potentiostat), while the wavelength was changed from 400 to 800 nm, with 25 nm steps. The light intensity was measured on a power meter (ILT 1400, International Light Technologies), and the observed photocurrent was normalized by the measured intensity at each wavelength.

### Characterization techniques

XRD patterns of the samples were collected using a Panalytical Empyrean X-ray diffractometer using Cu Kα radiation. Measurements were taken in a *θ*–2*θ* configuration, from 10° to 90° with a step size of 0.008. Scanning electron microscopy pictures were taken with a XL30 FEG microscope. Ultraviolet–visible spectra were recorded using a Varian Cary 50 Bio Ultraviolet-Visible spectrometer at a scanning rate of 300 nm min^−1^.

### Data availability

The raw data that support the findings of this study are available from the University of Cambridge data repository, http://dx.doi.org/10.17863/CAM.670

## Additional information

**How to cite this article:** Crespo-Quesada, M. *et al*. Metal-encapsulated organolead halide perovskite photocathode for solar-driven hydrogen evolution in water. *Nat. Commun.* 7:12555 doi: 10.1038/ncomms12555 (2016).

## Supplementary Material

Supplementary Information Supplementary Figures 1-18 and Supplementary Discussion

Supplementary Movie 1 Hydrogen evolution on a typical perovskite-based photocathode upon illumination (1 sun) in an aqueous solution (0.1 M borate, pH 8.5) under a nitrogen atmosphere and at room temperature.
