# Supplementary material for: Metal-encapsulated organolead halide perovskite photocathode for solar-driven hydrogen evolution in water
Source: Nat Commun. 2016 Sep 6;7:12555. doi: 10.1038/ncomms12555 (PMC5025836; doi:10.1038/ncomms12555)
Supplement: Supplementary Information — Supplementary Figures 1-18 and Supplementary Discussion [file ncomms12555-s1.pdf]

Supplementary Figures

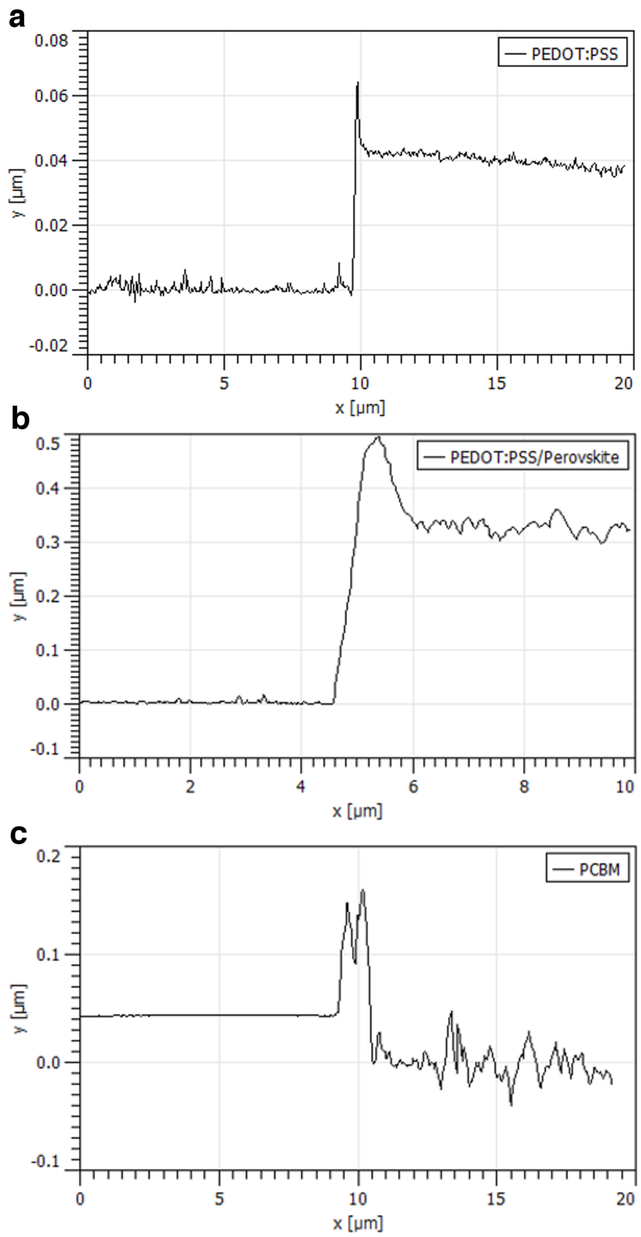

Supplementary Figure 1. AFM profiles of the charge transport and perovskite layers. AFM Image showing the thickness (*y* axis) of the layer with respect to the horizontal position of the AFM probe along the sample (*x* axis) for the a) PEDOT:PSS, b) PEDOT:PSS/perovskite and c) PCBM layers of the solar cells and photocathodes.

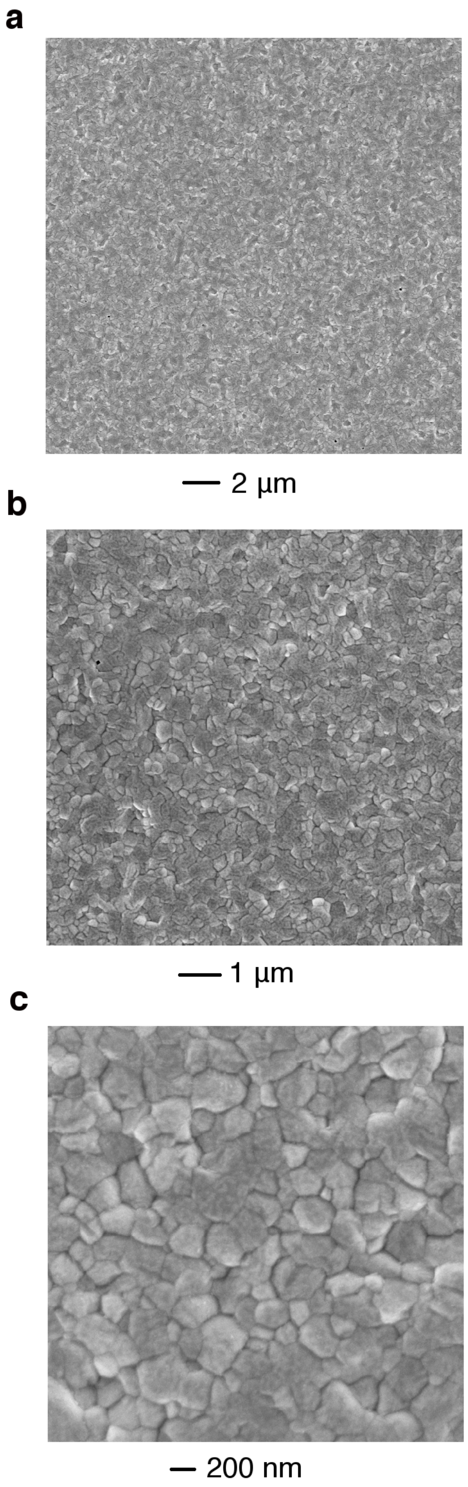

Supplementary Figure 2. SEM images of $CH_3NH_3PbI_3$ after solvent annealing. SEM images at different magnifications of 300 nm thick perovskite films on glass/FTO/PEDOT:PSS.

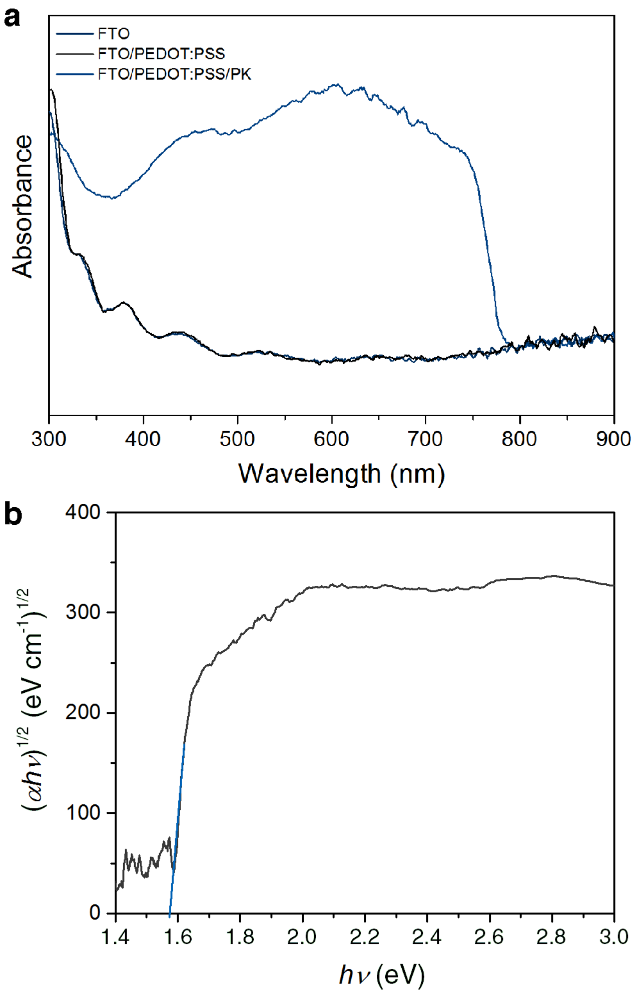

Supplementary Figure 3. UV-Vis spectra of the perovskite films. a) UV-Vis spectra of FTO, FTO/PEDOT:PSS and solvent annealed FTO/PEDOT:PSS/perovskite (PK), b) Tauc plot; $(\alpha h\nu)^{1/2}$ as a function of photon energy $h\nu$, where ν is the photon frequency, indicating the optical bandgap for the perovskite layer (1.56 eV).

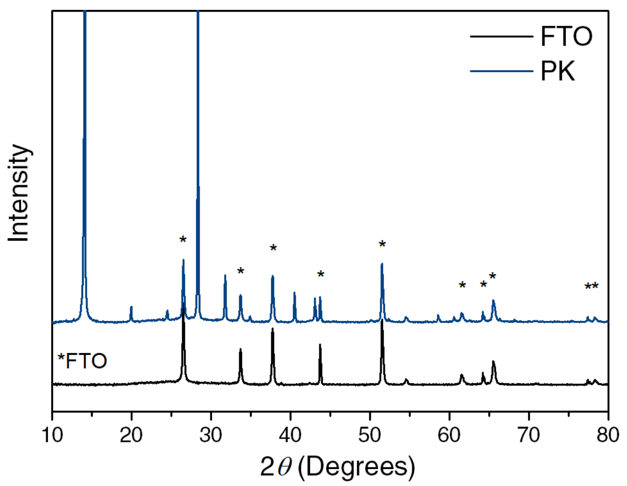

Supplementary Figure 4. XRD pattern of perovskite film. XRD pattern of the solvent annealed perovskite (PK) film and of unmodified FTO-coated glass.

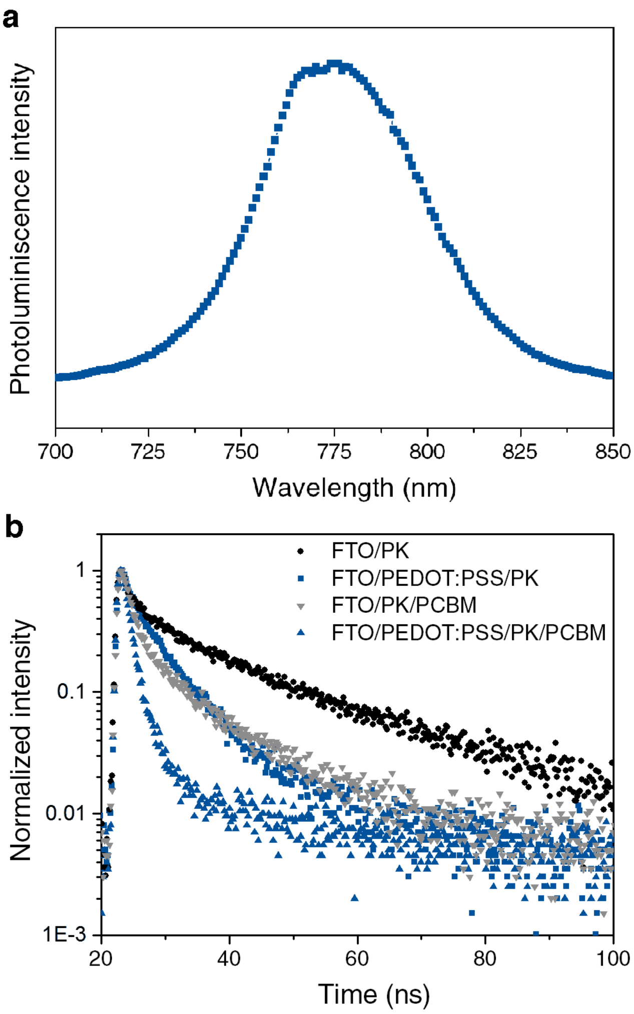

Supplementary Figure 5. Photoluminescence (PL) measurements. a) Steady-state PL emission and (b) time-resolved photoluminescence (PL) spectrum (λ = 775 nm) for perovskite (PK), PEDOT:PSS/PK, PK/PCBM and PEDOT:PSS/PK/PCBM layers deposited on FTO substrates. Excitation wavelength: 450 nm.

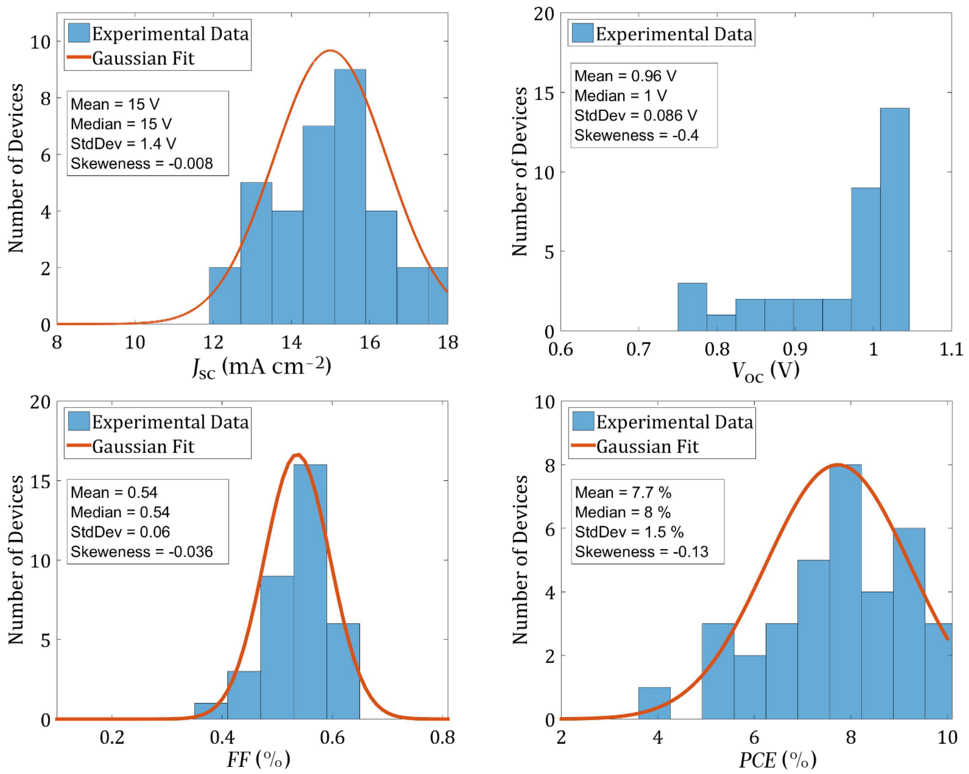

Supplementary Figure 6. Solar cell performance. Short circuit current (J_{sc}), open circuit voltage (V_{oc}), fill factor (*FF*) and power conversion efficiency (*PEC*) of the perovskite solar cells.

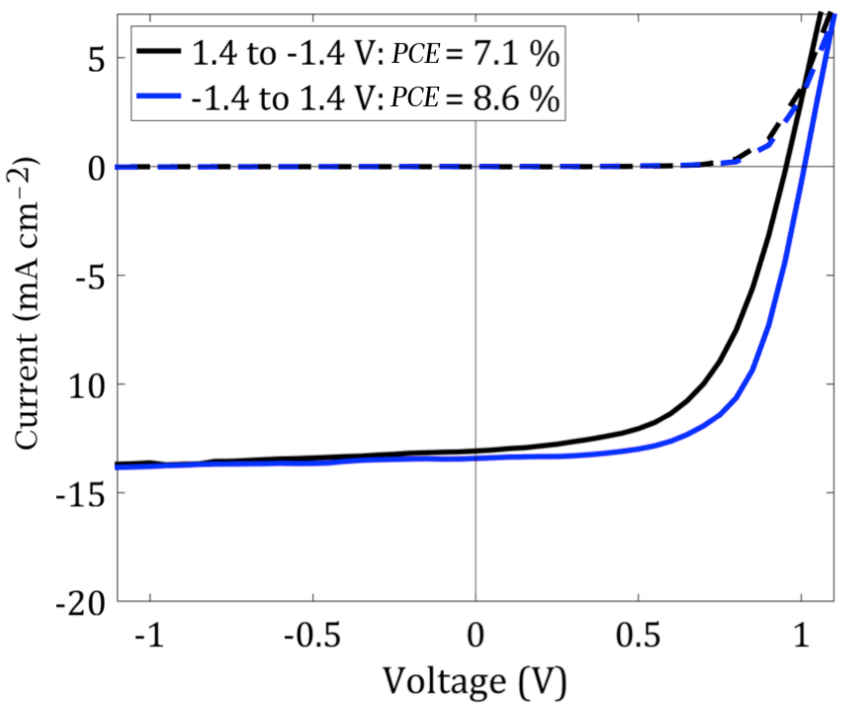

Supplementary Figure 7. Solar cell hysteresis. *IV* measurements performed in both bias directions: from reverse to forward (blue line) bias and *vice versa* (black line) in the dark (dashed line) and under illumination (solid line).

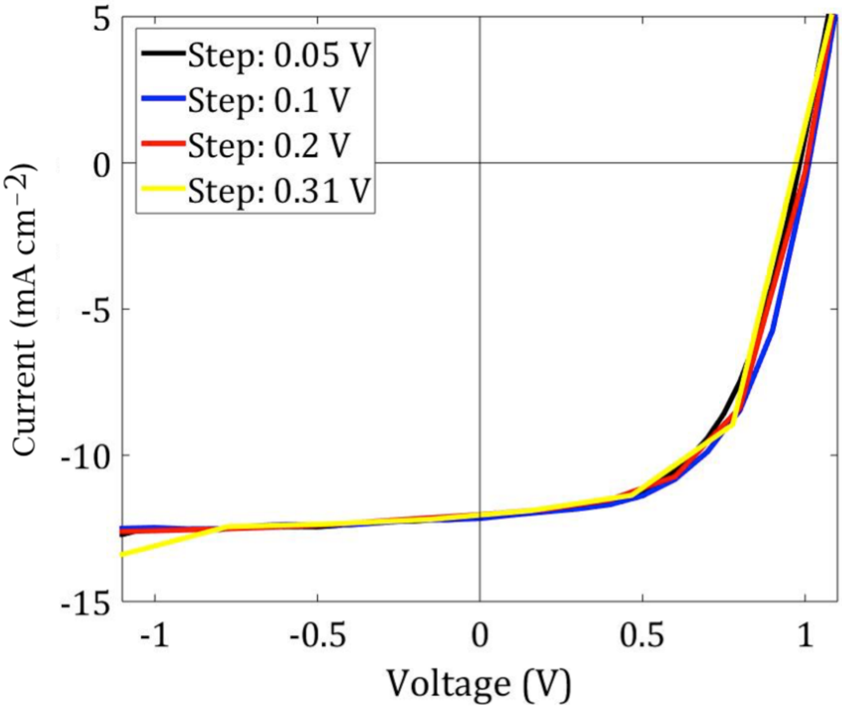

Supplementary Figure 8. Effect of step size on *IV* measurements. *IV* measurements performed with different step sizes.

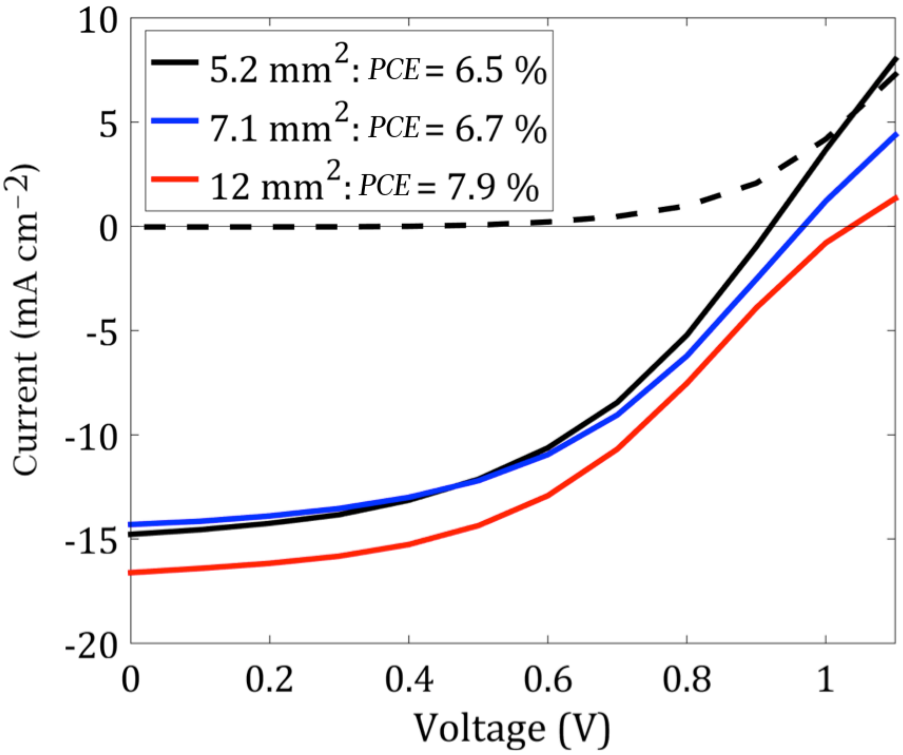

Supplementary Figure 9. Mask size dependency on *IV* measurements. *IV* measurements performed with differently sized masks.

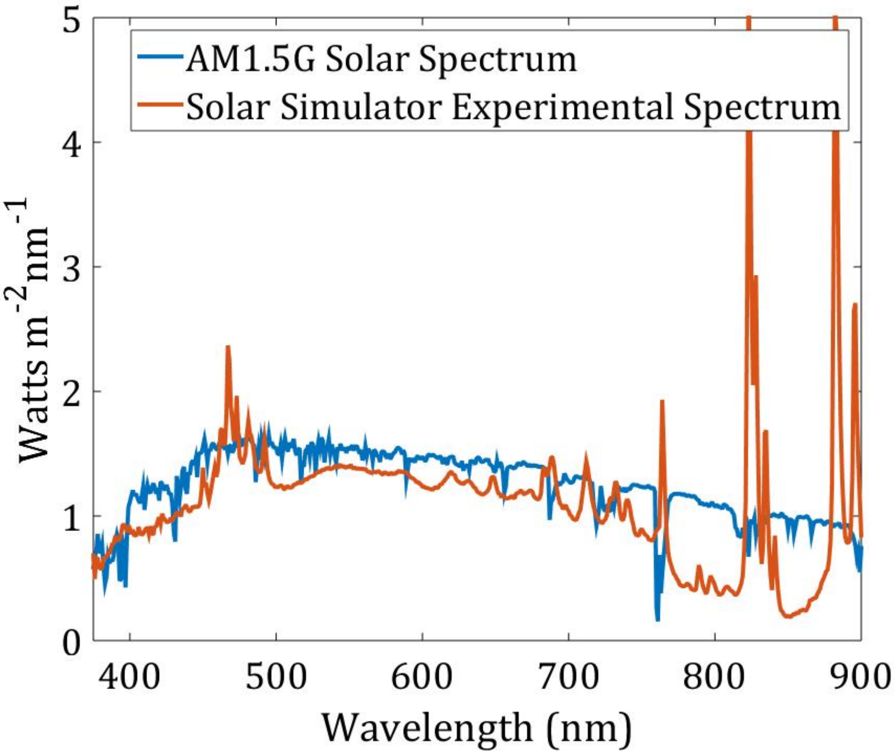

Supplementary Figure 10. Mismatch factor. Comparison between the solar simulator experimental solar spectrum and the AM1.5G standard spectrum.

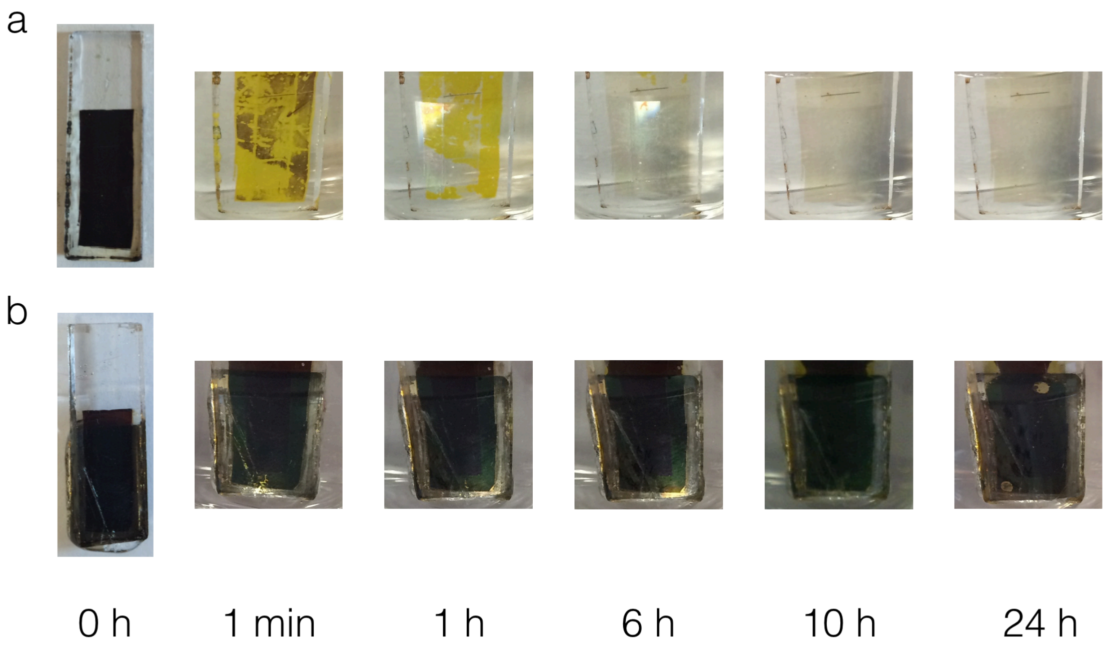

Supplementary Figure 11. Water resistance of the photocathodes. Water resistance of (a) an unprotected photocathode (PEDOT:PSS/$CH_3NH_3PbI_3$/PCBM/PEIE) and (b) protected photocathode (PEDOT:PSS/$CH_3NH_3PbI_3$/PCBM/PEIE:Ag/FM) submerged in aqueous buffer (0.1 M borate, pH 8.5) for 24 h in the dark without applied electrochemical bias.

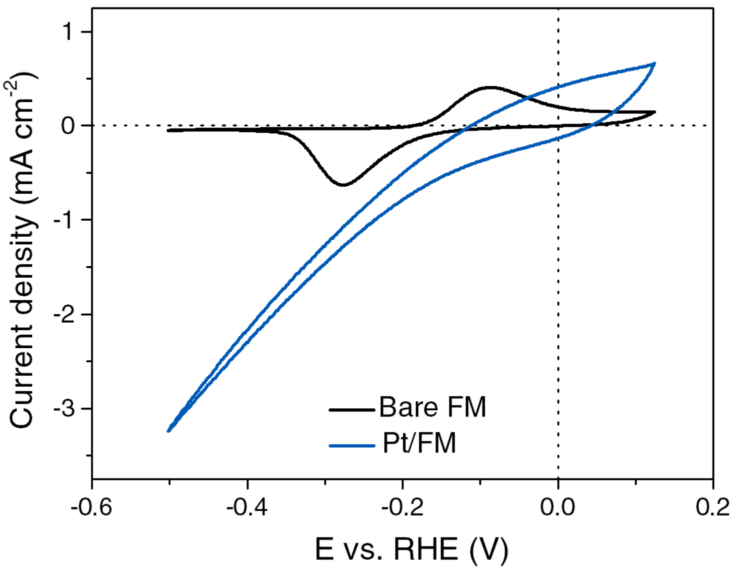

Supplementary Figure 12. Electrocatalytic H_2 evolution on Field's Metal. Cyclic voltammograms of bare FM and platinised FM under N_2 in a aqueous buffer solution (0.1 M borate, pH 8.5) at a scan rate of 100 mV s^{-1}.

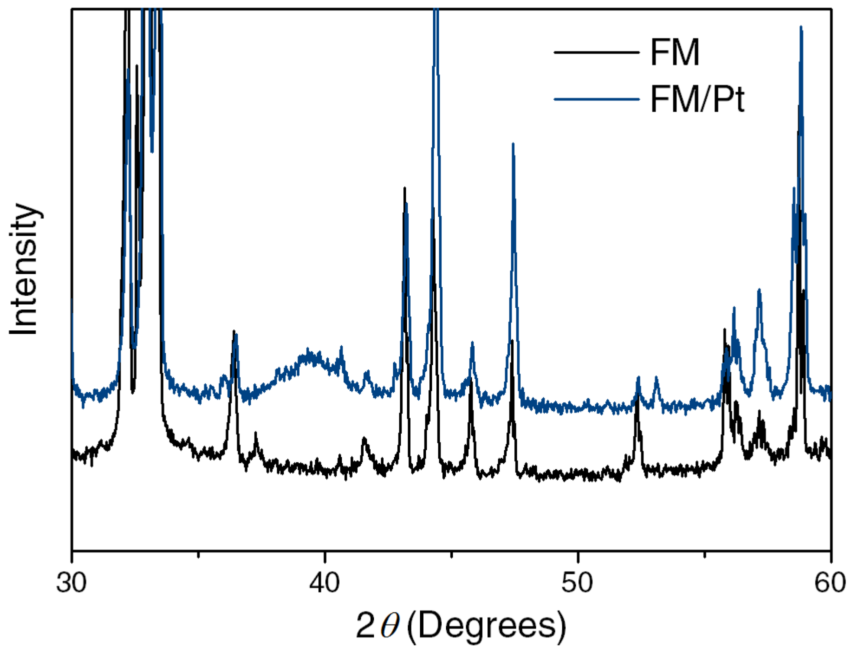

Supplementary Figure 13. XRD pattern of bare and platinized Field's metal. XRD pattern of bare and platinized FM.

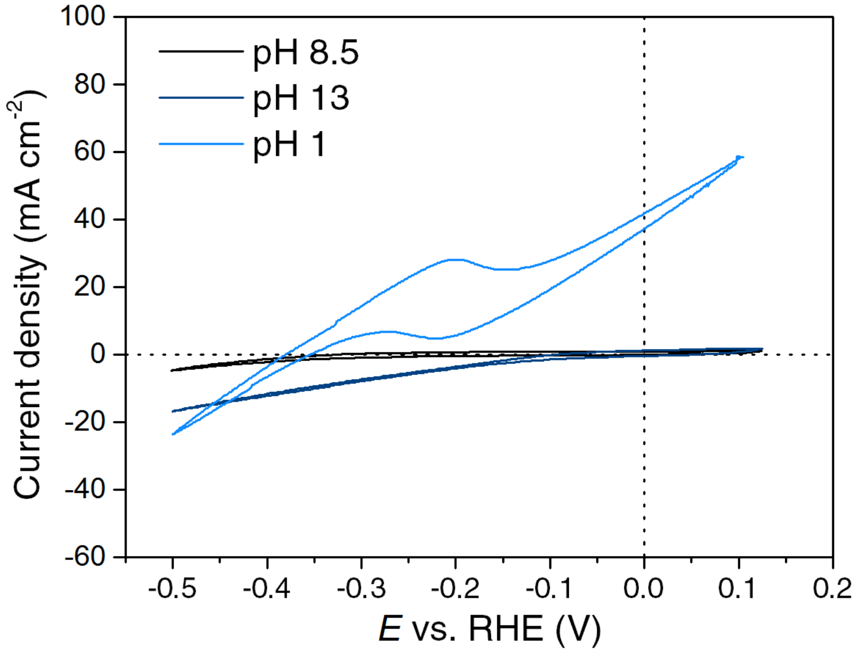

Supplementary Figure 14. Effect of pH on electrocatalytic proton reduction on Pt/FM. Cyclic voltammograms of platinised FM under N_2 in a 0.1 M KCl solution at pH 1, 8.5 and 13 at a scan rate of 100 mV s^{-1}.

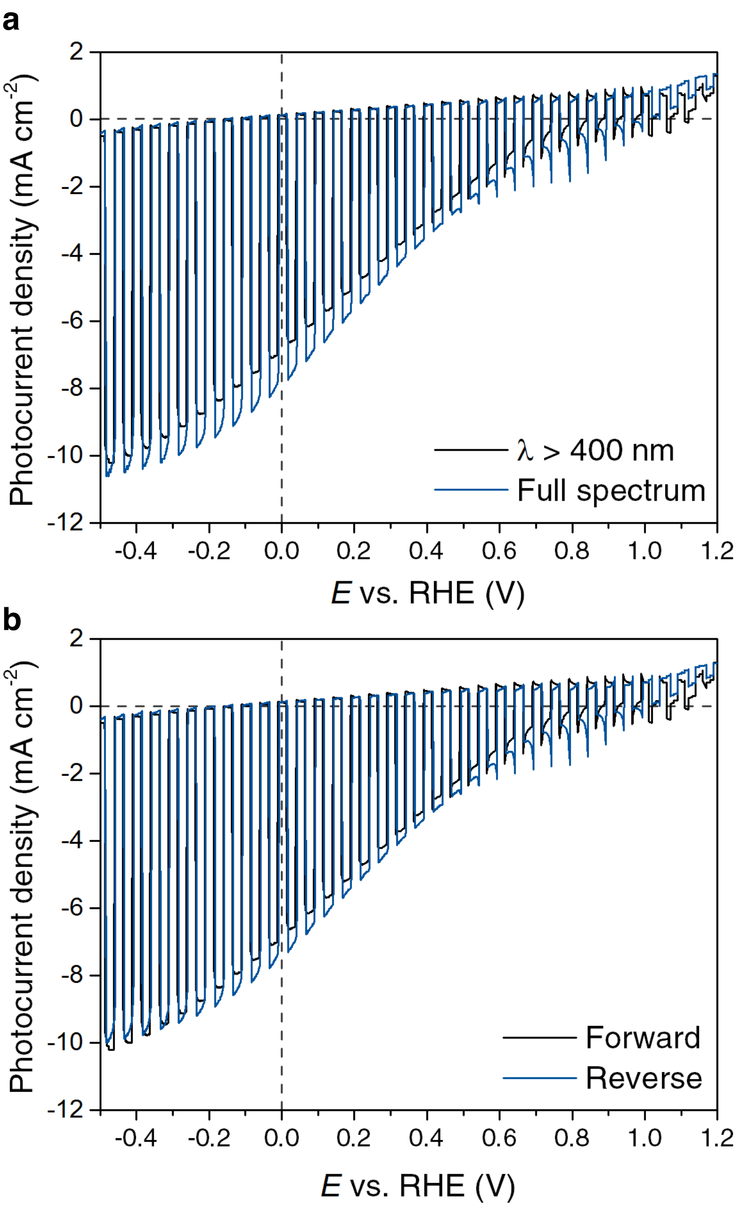

Supplementary Figure 15. Perovskite-based photocathodes for the hydrogen evolution reaction. Linear sweep voltammogram of the perovskite-based photocathode in (a) reverse and forward scans and (b) with and without a $\lambda > 400$ nm cut-off filter. Conditions: Aqueous electrolyte solution (0.1 M borate, pH 8.5, N_2 atmosphere) under chopped simulated solar light irradiation (100 mW cm^{-2}, AM 1.5G, $\lambda > 400$ nm) at a scan rate of 5 mV s^{-1}, room temperature.

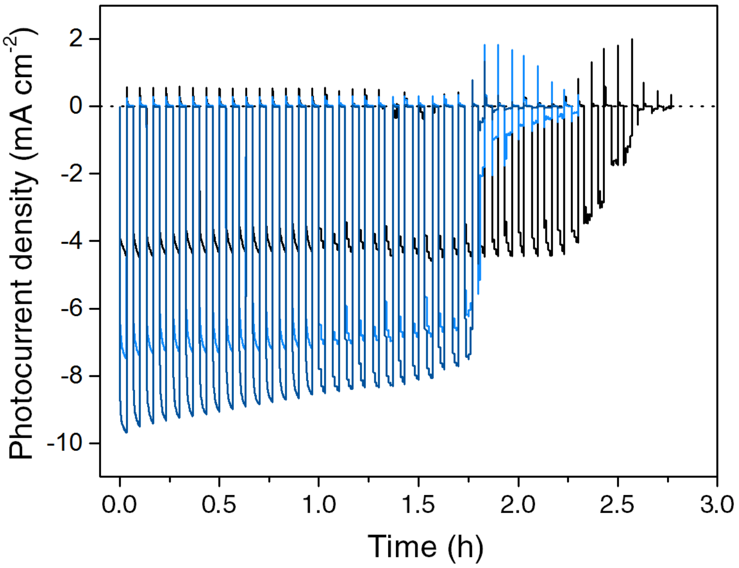

Supplementary Figure 16. Dependency of time to full degradation on photocurrent density*.* Effect of current-density in the stability of several perovskite-based photocathodes in an aqueous buffer solution (0.1 M borate, pH 8.5, N_2 atmosphere) under chopped simulated solar light irradiation (100 mW cm^{-2}, AM 1.5G, λ > 400 nm) at a scan rate of 5 mV s^{-1}, room temperature.

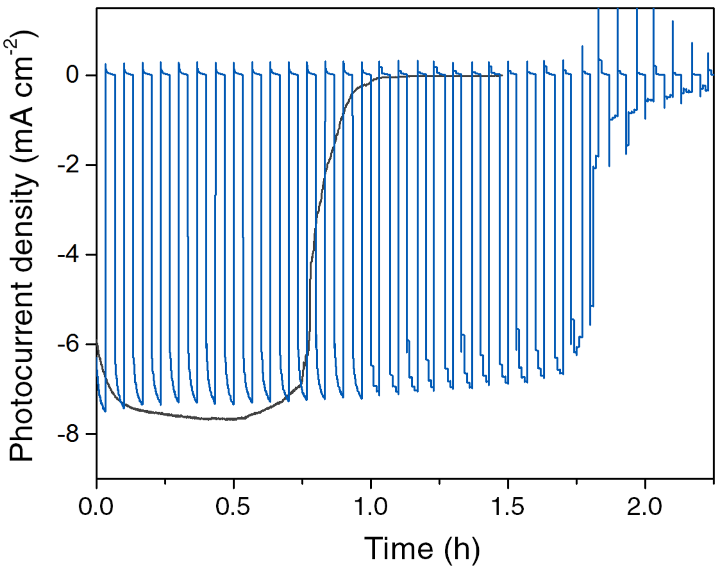

Supplementary Figure 17. Chronoamperometry under continuous illumination*.* Chronoamperometry of a typical photocathode under both chopped (blue line) and continuous (grey line) illumination in an aqueous buffer solution (0.1 M borate, pH 8.5, N_2 atmosphere, 100 mW cm^{-2}, AM 1.5G, $\lambda > 400$ nm) at a scan rate of 5 mV s^{-1}, room temperature.

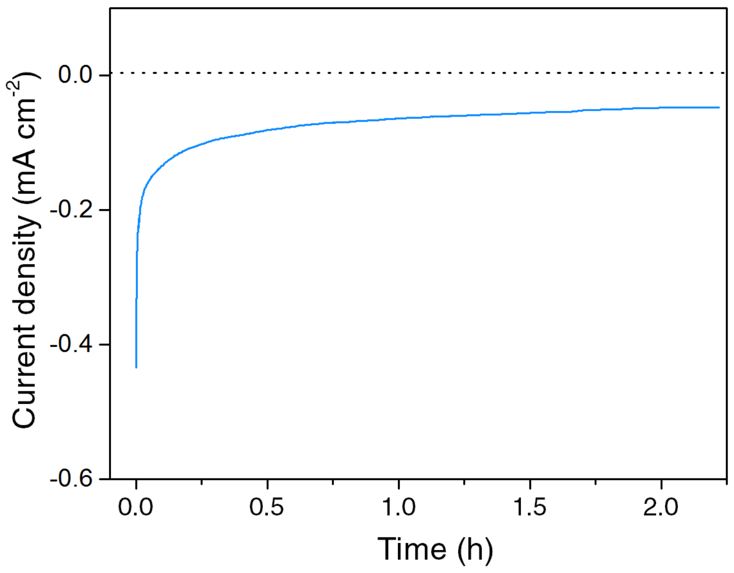

Supplementary Figure 18. Chronoamperometry of Pt/FM. Chronoamperometry of platinised FM under N_2 in aqueous buffer solution (0.1 M borate, pH 8.5) at an applied bias of –0.1 V versus RHE.

Supplementary Discussion

Solar cell performance and statistical analysis

The prepared solar cells were measured in a solar light simulator that provides about 90% of AM1.5G fluence over the absorption wavelength range of the perovskite material. This mismatch was taken into account when comparing the photocurrents obtained with the integrated photocurrent density obtained from *EQE* measurements. 35 working devices were measured and showed a short circuit current (J_{sc}), open circuit voltage (V_{oc}), fill factor (*FF*) and efficiency (η) of –15.0 ± 1.4 mA cm^{-2}, 1.0 ± 0.09 V, 54.0 ± 6.0 % and 7.7 ± 1.5 %, respectively (Supplementary Figure 6).

The mean, median, standard deviation and skewness for each of these parameters can be found on the figure. It is worth noting the relatively high negative skewness of the values obtained for the V_{OC} due to the closeness of the mean value to its physical limit.

Hysteresis

IV measurements were performed in both bias directions: from reverse to forward bias and *vice versa* (Supplementary Figure 7). No clear hysteresis effect was observed, beyond a slight improvement when measuring from reverse to forward (–1.4 V to 1.4 V), potentially due to an improved ion arrangement at the electrode interfaces.

Scan rate dependency

The *I-V* characteristics of the solar cells did not vary with the scan rate, as it can be seen in Supplementary Figure 8.

Mask size dependency

As shown in Supplementary Figure 9, there is a small dependency on mask size on the IV characteristics of the solar cells. We believe this to be attributed to an edge effect, which becomes more pronounced with the smaller masks.

Mismatch factor

In Supplementary Figure 10 we compare the spectrum as measured in our solar light simulator (Oriel 92250A) with a spectrometer after calibration with a reference silicon diode, with the solar spectrum standard (AM 1.5G). The external quantum efficiency measurements (*EQE*) were performed under illumination from a xenon lamp, which was passed through a monochromator (Oriel Cornerstone 260) before reaching the solar cell. The solar cell was then measured in short circuit. The current produced was compared to the current of a NIST-traceable calibrated photodiode (Thorlabs SM05-CAL). A reference diode (Thorlabs SM05) was used for accounting for changes in light intensity between measurements. The measured *EQE* was then used to predict the short-circuit current expected under AM 1.5 G and our solar simulator. The ratio between these two is the mismatch factor.
